# Effects of multi-site non-invasive brain stimulation on cognitive impairment after stroke: a systematic review and meta-analysis

**DOI:** 10.3389/fnhum.2025.1583566

**Published:** 2025-06-30

**Authors:** Heling Wang, Di Zhang, Xu Wang, Qixin Ding, Shenhong Ma, Qiaohua Han, Yuefang Li, Tianshu Li, Ying Li, Wanyue Li, Weisheng Zhuang

**Affiliations:** ^1^School of Rehabilitation Medicine, Henan University of Chinese Medicine, Zhengzhou, China; ^2^School of Clinical Medicine, Henan University, Zhengzhou, China; ^3^Zhengzhou First People’s Hospital, Zhengzhou, China; ^4^Department of Rehabilitation Medicine, Henan Provincial People’s Hospital, Zhengzhou, China

**Keywords:** MS-NIBS, stroke, cognition function, rehabilitation, meta-analysis

## Abstract

**Objective:**

Post-stroke cognitive impairment (PSCI) is one of the core symptoms following a stroke, which severely affects the prognosis of patients. This systematic review and meta-analysis aim to explore the effectiveness and safety of multi-site non-invasive brain stimulation (MS-NIBS) in enhancing the cognitive function of PSCI patients.

**Methods:**

A comprehensive search was conducted in multiple databases, including MEDLINE (PubMed), Embase, Web of Science, China National Knowledge Infrastructure (CNKI), Wanfang Data, VIP Database for Chinese Technical Periodicals, and Chinese Biomedical Literature Database (CBM). The search was performed up to 18 January 2025. The inclusion criteria for this meta-analysis were randomized controlled trials (RCTs) of MS-NIBS for PSCI. The primary outcome measure was the change in the global cognitive scale, while the secondary outcomes focused on improvements in attention, memory, visuospatial perception, and activities of daily living. The Cochrane Risk of Bias Tool was used to assess the quality of each eligible study. Meta-analysis and bias analysis were performed using RevMan (Version 5.3).

**Results:**

A total of 6 RCTs involving 416 samples were included in this paper. The findings from the primary outcomes revealed that the MS-NIBS group had significantly higher scores on the Montreal Cognitive Assessment (MOCA) of the cognitive composite scale (MD = 1.84, 95% CI = 1.21–2.48, *p* < 0.00001, *I*^2^ = 36%) compared to the single-site non-invasive brain stimulation (SS-NIBS) group. As for the secondary outcome measures, as shown by the Digit Span Test (DST) forward recall (MD = 0.94, 95% CI = −1.11 to 2.98, *p* = 0.37, *I*^2^ = 97%), DST backward recall (MD = 0.03, 95% CI = −0.24 to 0.29, *p* = 0.85, *I*^2^ = 0%), Clock Drawing Test (CDT) (MD = 1.65, 95% CI = 0.77–2.53, *p* = 0.0003, *I*^2^ = 54%), Trail Making Test (TMT) (MD = 4.2, 95% CI = 2.71–5.69, *p* < 0.00001, *I*^2^ = 14%), and Modified Barthel Index (MBI) for activities of daily living assessment (MD = 3.71, 95% CI = −4.77 to 12.20, *p* = 0.39, *I*^2^ = 75%), the MS-NIBS group showed improvements in visuospatial and trail-making test abilities. Subgroup analysis of the main outcome demonstrated that multi-site transcranial magnetic stimulation (MS-TMS) (MD = 2.1, 95% CI = 1.38–2.81, *p* < 0.00001, *I*^2^ = 48%) and the combined treatment of TMS and transcranial direct current stimulation (tDCS) (MD = 1.91, 95% CI = 0.81–3.01, *p* = 0.0007, *I*^2^ = 0%) exhibited superior efficacy compared to SS-NIBS.

**Conclusion:**

This meta-analysis provides evidence supporting that MS-NIBS, as an emerging neuromodulatory tool, is superior to SS-NIBS in improving the overall cognitive abilities of stroke patients. However, given the limited number of included studies, it is necessary to further validate these findings through large-scale, multi-center, double-blind, and high-quality RCTs.

**Systematic review registration:**

https://www.crd.york.ac.uk/prospero/, CRD42025640015.

## 1 Introduction

Stroke is a common cerebrovascular disease. The newly published Global Burden of Disease report shows that the annual prevalence of stroke among the Chinese population is 2,022 per 100,000 the annual incidence is 276.7 per 100,000 and the crude mortality rate is 153.9 per 100,000 (Ma et al., 2021). Among them, approximately half of the patients experience cognitive impairment to varying degrees (Huang et al., 2022). The mortality rate of patients with post-stroke cognitive impairment (PSCI) is significantly higher than that of patients with ordinary stroke. Moreover, the disability rate increases, which severely affects the patients’ quality of life and health status, imposing a heavy economic burden on families and society (Dowling et al., 2024; Ma et al., 2024). At present, there are no approved drug therapies specifically for PSCI or dementia. Non-drug therapies such as cognitive rehabilitation, psychological intervention, physical exercise, and acupuncture have shown unsatisfactory therapeutic effects (El Husseini et al., 2023). Non-invasive brain stimulation (NIBS), as an emerging treatment method, has been shown to potentially improve the cognitive function of patients with PSCI (Hara et al., 2021).

Transcranial magnetic stimulation (TMS) and transcranial direct current stimulation (tDCS) are two of the most representative methods within NIBS (Tereshin et al., 2022; Wang et al., 2022). TMS utilizes the pulsed magnetic field generated by a coil to selectively enhance or inhibit the excitability of the cerebral cortex. This alters the activity of neurons and regulates the functional connectivity within the brain network, thus influencing cognition (Lefaucheur et al., 2020; Valero-Cabré et al., 2017). Similarly, tDCS can also induce excitatory or inhibitory effects. Specifically, anodal tDCS can increase the excitability of the cerebral cortex in the target area, while cathodal tDCS produces an inhibitory effect (Woods et al., 2016). These effects, in turn, trigger changes in synaptic plasticity, such as inducing long-term potentiation and long-term depression, enhancing neural plasticity, and thus influencing cognition (Shepherd and Huganir, 2007).

Current evidence indicates that single-pulse TMS (including paired-pulse paradigms) serves as a diagnostic tool for investigating brain function, whereas repetitive TMS (rTMS) is employed to induce neuroplastic changes that persist beyond the stimulation period (Klomjai et al., 2015). Although a Large number of current studies have shown that both rTMS and tDCS can improve the cognitive function of patients with PSCI (Gao et al., 2023; Chen et al., 2024), conflicting results have also been reported in some studies (Park et al., 2013; Fregni et al., 2006; Kim et al., 2010). Search for effective neuromodulatory strategies to treat PSCI is still in the exploratory stage. Single-site non-invasive brain stimulation (SS-NIBS) refers to a neural modulation method that applies stimulation to a single target in the brain region through non-invasive techniques such as TMS, tDCS, and transcranial alternating current stimulation (tACS). Each stimulation only acts on a predefined brain region [such as the left DLPFC or the primary motor cortex (M1)]. It can only change local neural activities (such as cortical excitability), rather than the interactions within the brain network. Multi-site non-invasive brain stimulation (MS-NIBS), by contrast, can combine various stimulation methods such as TMS, tDCS, and tACS. It can regulate multiple brain regions by stimulating the neural activities in these regions simultaneously or in sequence (Guo et al., 2022). It is a non-invasive neural modulation technique for regulating the functions of distributed neural networks. This technique overcomes the limitations of single-site stimulation and brings new hope to neural modulation.

In previous studies, brain-stimulation techniques typically focused on a single target brain region. However, brain regions do not operate in isolation but work in concert as a network (Sporns et al., 2004). Therefore, the use of MS-NIBS to act on a network rather than a single brain region is gaining increasing attention (Fischer et al., 2017). To this end, several strategies have been proposed: a. Sequential single- modality stimulation strategy, such as cerebellar-cerebral tDCS (Grimaldi et al., 2014). b. Synchronous single-modality stimulation strategy, for example, using multiple electrodes in network tDCS electrode combinations (Fischer et al., 2017). c. Simultaneous dual-modality stimulation strategy, like applying 10 Hz rTMS to the primary motor cortex (iM1) and cathodal tDCS to the primary motor cortex (cM1) (Cho et al., 2017); d. Oscillatory stimulation strategy, such as dual-site transcranial alternating-current stimulation (tACS) to regulate inter-regional phase synchronization (Polanía et al., 2012); e. Cortico-cortical paired associative stimulation (cc-PAS) strategy to modulate cortical excitability and behavior (Rizzo et al., 2009). Multi-site stimulation may have the potential to simultaneously promote recovery in multiple domains and their interaction even super-additive effects.

Multi-site non-invasive brain stimulation, which emerges as a novel therapeutic modality, has exhibited preliminary efficacy in stroke rehabilitation, depression treatment, and cognitive enhancement (Ren et al., 2024; Lefaucheur et al., 2020; Valiengo et al., 2013). In stroke rehabilitation, research has revealed that the bilateral rTMS group demonstrated significantly more substantial improvement in the Brunnstrom Recovery Stage compared to the 10 Hz rTMS group (Sasaki et al., 2014). In the context of depression, research has shown that the sequential application of both high-frequency left-side rTMS and low-frequency rTMS to the right prefrontal cortex exhibits substantial treatment efficacy in patients with treatment-resistant major depression. The treatment response accumulates to a clinically significant level over a 4 to 6 week course of active treatment (Fitzgerald et al., 2006). In terms of cognitive enhancement, research indicates that 10 Hz rTMS was applied to the frontal and parietal targets within the cognitive attention network (cingulo-frontal-parietal, CFP) of the subjects, and then the subjects underwent functional magnetic resonance imaging (fMRI) examination. As a result, activation of varying degrees was observed in the brain regions related to cognition (Feng et al., 2019). Zhang et al. (2019) selected the left DLPFC and the left temporal lobe as the stimulation targets for patients with Alzheimer’s disease (AD). The results showed that the cognitive function and emotional state of the patients were improved to varying degrees. However, there is currently no meta-analysis evaluating the effectiveness of MS-NIBS in the treatment of PSCI. Therefore, the aim of this study was to conduct a meta-analysis to evaluate the impact of MS-NIBS on PSCI.

## 2 Data and methods

Due to the fact that this study was a systematic review of previously published studies, neither patient consent nor ethical approval was necessary (Higgins and Thompson, 2002). Based on the Preferred Reporting Items for Systematic Reviews and Meta-Analyses guidelines (PRISMA) and previously published protocols, this meta-analysis was carried out (Moher et al., 2009). Details of the protocol used to perform this system evaluation have been registered with PROSPERO (reference number: CRD42025640015).

### 2.1 Search strategy

In accordance with the PRISMA statement, searches were conducted in databases such as MEDLINE (via PubMed), Embase, Web of Science, CNKI, Wanfang, VIP, and the CBM. The search was conducted up to 18 January 2025, without language restrictions. Keywords used included “stroke,” “cognitive function,” “transcranial direct current stimulation,” “transcranial magnetic stimulation,” etc., the specific search strategy can be found in the Supplementary material.

### 2.2 Inclusion and exclusion criteria

Inclusion criteria: (1) Patients with PSCI were treated; (2) the intervention method of the experimental group was MS-NIBS, MS-NIBS involves stimulation of ≥ 2 brain regions and common modalities (rTMS, tDCS); (3) the intervention method of the control group was single-site or sham NIBS; (4) the outcome measures in this paper are the efficacy of MS-NIBS in the treatment of PSCI. the main index is the MOCA score of cognition after treatment with different stimulation methods, and the secondary indexes are the scores of the Digit Span Test (DST), Clock Drawing Test (CDT), Trail Making Test (TMT), and Modified Barthel Index (MBI); (5) randomized controlled trial (RCT). Exclusion criteria: (1) insufficient data; (2) publications; (3) the literature with original data still cannot be found after trying all methods; (4) Poor study quality (PEDro score < 5).

### 2.3 Data extraction

Data extraction was conducted individually by both researchers (HW and DZ), and disputes were resolved by a third researcher (XW) when they arose. The data included information such as study design, sample size, patient characteristics (age, gender, stroke duration, lesion location, stage), treatment regimens (frequency, intensity, number of pulses, intervention time, stimulation targets), and outcome measures. If data were missing or unclear, attempts were made to contact the authors to obtain them. When evaluating multiple cognitive function assessment scales, data from commonly used scales such as the MoCA or MMSE were prioritized for analysis. Data extraction focused on direct retrieval of reported outcomes (e.g., MMSE, MoCA scores) and statistical parameters (mean, standard deviation, sample size), as most studies utilized standardized measures compatible with meta-analysis without requiring additional data transformation.

### 2.4 Quality assessment

In this study, to ensure the reliability and scientific nature of the included studies, the Physiotherapy Evidence Database (PEDro) scale was used to assess the methodological quality of each included RCT. The PEDro scale is a professional scale consisting of 11 items and is widely applied in the field of methodological quality scoring for RCTs (Cashin and McAuley, 2020). When using this scale for assessment, except for the first item which is judged as either “YES” or “NO”, for other items related to internal validity, each item is awarded 1 point if the requirements are met, with a full score of 10 points. In the assessment of study quality, according to the generally recognized criteria (Teasell et al., 2003), if a study scores 4 points or more on the PEDro scale, it is regarded as a high-quality study. However, for those studies with a score of 6 points or more, but with scores of 2 or 3 points, respectively, for the two key criteria of randomization and concealed allocation, they will be downgraded to medium-quality studies. Based on the above criteria, studies with poor quality (scoring less than 4 points) were excluded in this study to ensure the reliability and validity of the research results.

### 2.5 Statistical analysis

For studies that used the same scale to assess outcomes, the number of participants, means, and standard deviations (SDs) of the experimental and control groups before and after intervention were analyzed in RevMan 5.3. For continuous outcomes, if the measurement units were consistent across trials, the results were presented as the weighted mean difference (MD) with a 95% confidence interval (95% CI). If the scales were inconsistent, the standardized mean difference (SMD) with a 95% CI was used (Murad et al., 2019). When the meta-analysis involved more than 10 articles, a funnel plot was used to detect publication bias.

Sub-group analyses were conducted according to factors such as the type of NIBS (tDCS vs. rTMS) and scales. The Cochrane Q-test and Higgins’ *I*^2^ statistic were used to assess the heterogeneity among studies (Higgins and Thompson, 2002). An *I*^2^ value of less than 25% indicated low heterogeneity, 25% ≤ *I*^2^ ≤ 75% indicated moderate heterogeneity, and an *I*^2^ value greater than 75% indicated high heterogeneity (Higgins et al., 2003). When *I*^2^ < 50%, a fixed-effect model was adopted; otherwise, a random-effect model was used (Borenstein et al., 2017). Egger’s linear regression test and funnel-plot visualization were employed to evaluate publication bias (Egger et al., 1997). Sensitivity analysis was performed to explore the impact of excluding low-quality studies and cross-over design studies on the effect size. The significance level for all statistical analyses was set at *p* < 0.05. Finally, the effect sizes were classified as small (0.2), medium (0.2–0.8), and large (0.8).

## 3 Results

### 3.1 Study characteristics and methodological quality evaluation results

**FIGURE 1:**
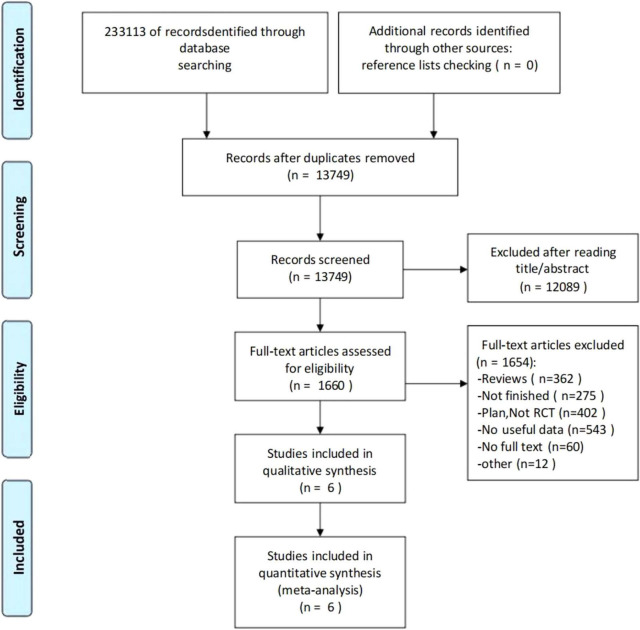
The flowchart of the literature search and screening process.

The screening flow chart is shown in Figure 1. A comprehensive search strategy identified 233,113 records from databases, with no additional records identified through other sources. After removal of duplicates, 13,749 records underwent title/abstract screening, of which 12,089 were excluded due to irrelevance to the research topic. Full-text assessment was performed for 1,660 articles, with 1,654 excluded for the following reasons: review articles (*n* = 362), ongoing/uncompleted studies (*n* = 275), study protocols/non-RCTs (*n* = 402), insufficient data (*n* = 543), unavailable full texts (*n* = 60), and unconvertible data formats (*n* = 12). Ultimately, 6 studies (Fang et al., 2022; Luo et al., 2022; Xu et al., 2022; Hu et al., 2023; Bai, 2024; Xu et al., 2024) (*N* = 416) met all eligibility criteria and were included in both qualitative synthesis and quantitative meta-analysis.

The characteristics of enrolled participants and all relevant information from studies meeting inclusion criteria are presented in Table 1. The study protocol incorporated NIBS under different conditions. In one study (Fang et al., 2022), the control group was a sham-stimulation group. In another study (Luo et al., 2022), the control group was an SS-NIBS group. In the remaining four studies (Xu et al., 2022; Hu et al., 2023; Bai, 2024; Xu et al., 2024), both an SS-NIBS group and a sham-stimulation group were included.

**TABLE 1:** Basic information of the included studies.

Reference	*N*	Age (year) [mean (SD)]	Gender [male (%)]	Time post stroke [mean (SD)]	Lesion side [right (%)]	Phase	Stimulation side	Parameter	Outcome messure
Bai, 2024	22	rTMS: 54.59 (14.696)	16 (72.7%)	38.5 (11.875) d	NR	Subacute	Left DLPFC	10 Hz 80% RMT; 1200 pulses; 5 times/w; 3 w	MMSE MoCA CDT DST CWT MBI
	22	SS-iTBS: 51 (13.259)	19 (86.4%)	22 (11.5) d	NR		Left DLPFC	5 Hz 80% RMT; 1200 pulses; 5 times/w; 3 w	
	22	MS-iTBS: 50.68 (13.185)	18 (81.8%)	40.5 (13.25) d	NR		Left DLPFC; Left prefrontal lobe; Broca	5 Hz 80% RMT; 1200 pulses; 5 times/w; 3 w	
	22	Sham: 53.73 (14.736)	14 (63.6%)	51.5 (13.625) d	NR		No	Rehabilitation	
Hu et al., 2023	12	rTMS: 63.87 (6.31)	12 (100%)	96.25 (29.11) d	NR	Chronic	Left DLPFC	5 Hz 80% RMT; 1200 pulses; 5 times/w; 4 w	MoCA
	10	rTMS-tDCS: 64.49 (7.15)	8 (80%)	95.2 (36.16) d	NR		AH: A (T5/T6); UH: C (PPC) (P3/P4) + rTMS	1.2 mA; 5 times/w; 4 w	
	12	Sham: 61.48 (9.08)	10 (83.3%)	105.83 (44.2) d	NR		No	Rehabilitation	
Xu et al., 2022	30	Sham: 57.25 (9.54)	18(60%)	22.86 (14.21) d	NR	Subacute	No	Rehabilitation	MoCA DST
	30	rTMS: 59.45 (8.32)	19 (63.3%)	24.05 (14.61) d	NR		5 HZ: PFC	5 Hz 80% RMT; 1000 pulses; 5 times/w; 4 w	
	30	rTMS: 58.55 (10.06)	19 (63.3%)	21.55 (11.56) d	NR		1 HZ (UH: PFC); 5 HZ (AH: PFC)	UH: 1 Hz + 5 Hz 80% RMT; 1000 pulses; 5 times/w; 4 w	
Luo et al., 2022	29	rTMS: 68.50 (4.18)	19 (65.52%)	85.68 (7.70) d	15 (51.7%)	Chronic	Left DLPFC ①	10 Hz 80% RMT; 800 pulses; 5 times/w; 8 w	MoCA TMT CWT
	30	tDCS: 67.29 (4.91)	18 (60%)	88.93 (5.32) d	15 (50%)		A (left DLPFC); C (shoulder)②	1.2 mA; 5 times/w; 8 w	
	28	rTMS + tDCS: 69.04 (5.12)	17 (60.7%)	90.74 (6.74) d	15 (53.6%)		① + ②	① + ② interval 10 min	
	29	tDCS + rTMS: 66.73 (4.16)	17 (58.6%)	79.60 (7.69) d	13 (44.8%)		② + ①	② + ①interval 10 min	
Fang et al., 2022	20	MS-tDCS: 63.25 (7.43)	17 (85%)	1.85 (0.93) m	20 (100%)	Subacute	A: p4; C: p3	0.6 mA; 5 times/w; 8 w	CDT MBI
	20	Sham: 61.80 (8.04)	16 (80%)	1.70 (1.08) m	20 (100%)		No	30 s stop	
Xu et al., 2024	18	Sham: 69.06 (8.94)	13 (72.2%)	75.42 (23.68) d	12 (66.7%)	Subacute	No	No	MoCA MBI TMT DST
	15	SS-rTMS: 67.53 (10.63)	12 (80.0%)	69.47 (27.84) d	8 (53.3%)		Left DLPFC	10 Hz 80% RMT; 2000 pulses; 5 times/w; 4 w	
	15	MS-rTMS: 67.20 (10.09)	7 (46.7%)	74.63 (24.21) d	4 (26.7%)		Left DLPFC + M1	M1: 10 Hz 80% RMT; 1200 pulses; 5 times/w; 4 w	

①left DLPFC, ②A(left DLPFC);C(shoulder).

The study interventions involved various types of NIBS. One study (Fang et al., 2022) applied tDCS, two studies (Xu et al., 2022; Xu et al., 2024) applied rTMS, and two studies (Luo et al., 2022; Hu et al., 2023) applied both tDCS and rTMS. Additionally, one study (Bai, 2024) applied MS-iTBS. Regarding the outcome measures of cognitive function, five studies (Luo et al., 2022; Xu et al., 2022; Hu et al., 2023; Bai, 2024; Xu et al., 2024) reported the MOCA, three studies (Xu et al., 2022; Bai, 2024; Xu et al., 2024) reported the DST, two studies (Fang et al., 2022; Xu et al., 2024) reported the CDT, two studies (Luo et al., 2022; Xu et al., 2024) reported the TMT, three studies (Fang et al., 2022; Bai, 2024; Xu et al., 2024) reported the MBI, and only one study (Bai, 2024) reported the MMSE.

**FIGURE 2:**
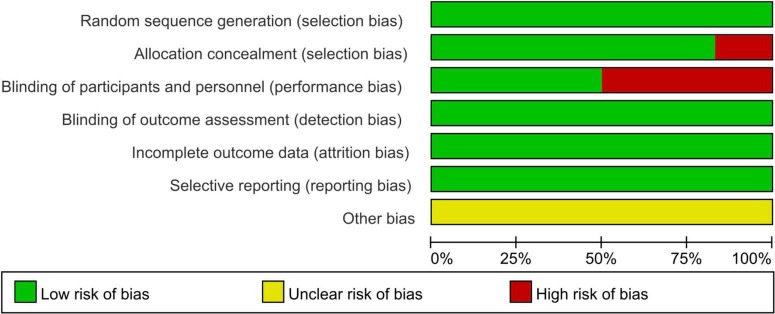
Risk of bias summary.

The risk of bias assessment unveiled that out of the 6 articles, Bai (2024), Fang et al. (2022) and Xu et al. (2022) did not follow the double-blind principle during the intervention, whereas the remaining papers exhibited high quality (Figure 2).

### 3.2 Adverse effects

In this study, all participants showed good tolerance to MS-NIBS, and no significant adverse events occurred. The researchers did not observe any related adverse reactions, and the patients did not report any discomfort. This indicates that MS-NIBS demonstrated high safety and tolerability in the application of this study, providing strong safety support for subsequent research and applications.

### 3.3 Quality assessment

Table 2 presents the methodological quality assessment of the included studies, which was evaluated using the PEDro scale. All the included studies scored above 4 on the PEDro scale, indicating adequate quality. The mean PEDro score was 6.67 (SD = 0.82), with a scoring range from 6 to 8.

**TABLE 2:** Methodological quality of included studies.

Reference	Eligibility criteria specified (Yes/No)	Random allocation (0/1)	Concealed allocation (0/1)	Comparable at baseline (0/1)	Blinded subjects (0/1)	Blinded assessors (0/1)	Adequate follow-up (0/1)	Intention to-treat analysis (0/1)	Between group comparisons (0/1)	Point estimates and variability (0/1)	Summary
Bai, 2024	Yes	1	0	1	0	0	1	1	1	1	7
Hu et al., 2023	Yes	1	0	1	0	0	1	1	1	1	7
Xu et al., 2022	Yes	1	0	1	0	0	1	0	1	1	6
Luo et al., 2022	Yes	1	0	1	0	0	1	0	1	1	6
Fang et al., 2022	Yes	1	0	1	0	0	1	0	1	1	6
Xu et al., 2024	Yes	1	1	1	1	1	0	1	1	1	8

### 3.4 Meta-analysis results

**FIGURE 3:**
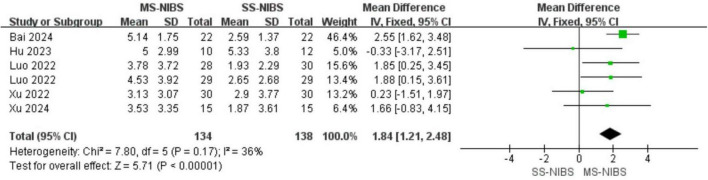
Forest plot for the meta-analysis comparing the MOCA of MS-NIBS versus SS-NIBS in treating PSCI.

By collecting post-intervention data from a total of 416 participants in 6 studies, the impact of MS-NIBS on PSCI, as compared with SS-NIBS, was evaluated. The pooled meta-analysis revealed that, in terms of overall cognitive function assessment, the MS-NIBS group had significantly higher scores on the comprehensive cognitive scale MOCA (MD = 1.84, 95% CI = 1.21–2.48, *p* < 0.00001, *I*^2^ = 36%) compared with the SS-NIBS group (Figure 3).

**FIGURE 4:**
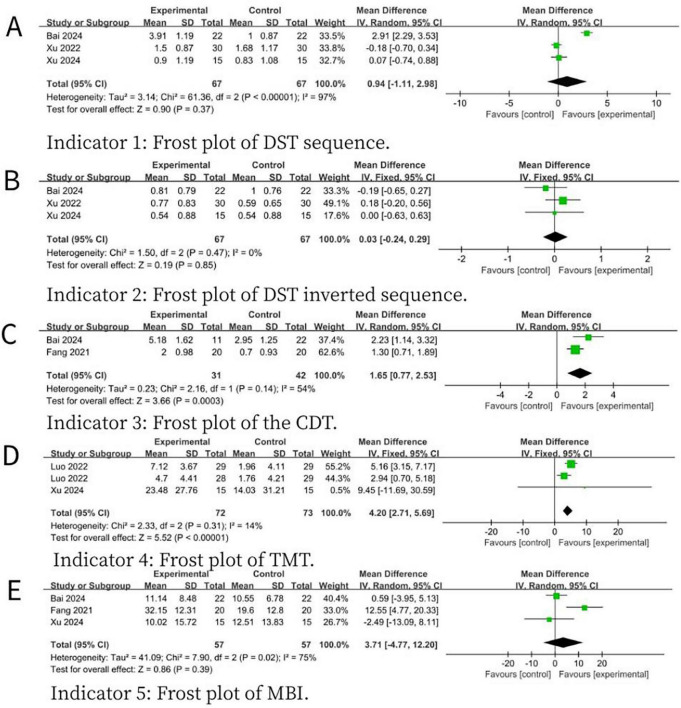
Forest plot for the meta-analysis comparing the scores of various scales of MS- NIBS versus SS-NIBS in treating PSCI. **(A)** Frost plot of DST sequence. **(B)** Frost plot of DST inverted sequence. **(C)** Frost plot of the CDT. **(D)** Frost plot of TMT. **(E)** Frost plot of MBI. DST sequence, digit span test forward recall; DST inverted sequence, digit span test backward recall; CDT, clock drawing test; TMT, trail making test; MBI, modified barthel index.

For secondary outcome measures, compared with SS-NIBS, MS-NIBS demonstrated improvements in visuospatial and trail-making test abilities, as shown in the following results: the forward recall of the DST (MD = 0.94, 95% CI = −1.11 to 2.98, *p* = 0.37, *I*^2^ = 97%) (Figure 4A), the backward recall of the DST (MD = 0.03, 95% CI = −0.24 to 0.29, *p* = 0.85, *I*^2^ = 0%) (Figure 4B), the CDT (MD = 1.65, 95% CI = 0.77–2.53, *p* = 0.0003, *I*^2^ = 54%) (Figure 4C), the TMT (MD = 4.2, 95% CI = 2.71–5.69, *p* < 0.00001, *I*^2^ = 14%) (Figure 4D), and the MBI (MD = 3.71, 95% CI = − 4.77 to 12.20, *p* = 0.39, *I*^2^ = 75%) (Figure 4E).

**FIGURE 5:**
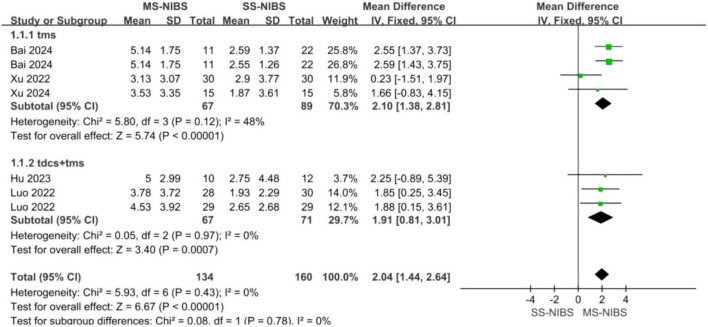
subgroup analysis of MOCA scores comparing DS-TMS and (tDCS + TMS).

Subgroup analysis of the primary outcomes indicated that MS-TMS (MD = 2.1, 95% CI = 1.38–2.81, *p* < 0.00001, *I*^2^ = 48%) and the combined treatment of TMS and tDCS (MD = 1.91, 95% CI = 0.81–3.01, *p* = 0.0007, *I*^2^ = 0%) demonstrated superior efficacy compared with SS-NIBS (Figure 5).

## 4 Discussion

This meta-analysis aimed to comprehensively analyze and summarize existing studies to evaluate the efficacy of MS-NIBS in treating PSCI. The research results demonstrated that, compared with SS-NIBS, MS-NIBS could significantly improve cognitive function. This is the first meta-analysis to comprehensively report the improvement effect of MS-NIBS on PSCI. The results showed that the MS-NIBS group was significantly superior to the SS-NIBS group in overall cognitive function. Sub-group analysis revealed that the MS-NIBS group was also superior to the SS-NIBS group in visuospatial and trail-making test abilities. Moreover, both the MS-TMS group and the MS-tDCS group were superior to the SS-NIBS group in overall cognition.

Cognitive impairment poses a significant challenge during the rehabilitation process of stroke patients. NIBS technology has been widely used to improve functional deficits following neuronal injury, and previous research (Khedr et al., 2012) has confirmed that it has achieved certain results in the treatment of some patients. However, the selection of stimulation parameters and stimulation targets plays a crucial role in the efficacy of stimulation therapies (Klomjai et al., 2015). Previous studies have shown that, compared with SS-NIBS, MS-NIBS has better efficacy (Wang et al., 2023; Bentwich et al., 2011; Lee et al., 2016; Long et al., 2018) shown in Figures 3, 4, the results of this study are reliable and consistent, strongly confirming the advantages of MS-NIBS. Even though there were differences in the type of NIBS, the time interval since stroke onset, and the treatment course, our trials yielded reliable and consistent results, verifying the benefits of MS-NIBS.

However, the specific mechanisms by which MS-NIBS treats PSCI remain unclear. Research indicates that after a stroke, various forms of neural network reorganization occur in both the ipsilateral and contralateral hemispheres (Eriksson et al., 2023), and functional recovery is associated with neuroplastic changes in the brain (Soleimani et al., 2023) changes include neurogenesis, gliogenesis, axonal sprouting, alterations in the excitatory/inhibitory balance, and so on. Many scientists have explored the relationship between cognitive recovery after stroke and cortical reorganization (Dacosta-Aguayo et al., 2014; Maja, 2013), revealing the importance of the inter-hemispheric activation balance in the cognitive-related cortex for cognitive recovery in stroke patients. MS-NIBS exerts its effects on PSCI patients based on this principle (Di Pino et al., 2014).

The included study (Bai, 2024) found that multi-site intermittent theta-burst stimulation (MS-iTBS) was superior to single-site iTBS (SS-iTBS) in improving PSCI. This can be attributed to the central role of neural connections between different brain regions in the neural network of the brain in the realization of cognitive function. MS-iTBS can enhance the connectivity of the brain network and optimize the efficiency and accuracy of information transmission. By stimulating brain regions such as the DLPFC, rostral marginal prefrontal cortex, and Broca’s area, the connections of brain networks like the frontoparietal network and limbic system can be strengthened, thereby improving cognitive function (Yin et al., 2020). Another study (Luo et al., 2022) showed that the stimulation sequence of applying tDCS first and then TMS had a better effect, outperforming the use of tDCS or TMS alone, as well as the sequence of applying TMS first and then tDCS. This is mainly due to the differences in the mechanisms of action of tDCS and TMS. TDCS regulates the activity of cerebral cortical neurons through direct current, while TMS affects neuroelectrophysiological activities by magnetic field stimulation. Applying tDCS first can change the resting membrane potential of the cerebral cortex, laying a more favorable neurophysiological foundation for the subsequent TMS stimulation and enhancing their synergistic effect (Lefaucheur et al., 2020).

At present, there are two theoretical models for the clinical application of neuromodulation in rehabilitation for stroke. They are the bilateral hemispheric competition model that advocates inhibition in the unaffected hemisphere (UH) or excitation in the affected hemisphere (AH) (Lefaucheur et al., 2020) and the vicariation model that advocates excitation in residual brain area of the AH or the UH (Di Pino et al., 2014). Given the inconsistency between these two theoretical models in guiding NIBS treatment, there is currently no consensus on the use of excitatory or inhibitory modulation in UH (Long et al., 2018). Therefore, in addition to these two models, a bimodal balance restoration model has also been proposed (Chen et al., 2023) model combines the advantages of the inter-hemispheric inhibitory competition and substitution models. It posits that post-stroke functional recovery depends not only on inter-hemispheric balance but also on the functional reorganization and compensation of the brain network. The model emphasizes that post-stroke recovery is a dynamic process involving the coordinated action of multiple brain regions. In a healthy brain, the neural activities of the bilateral cerebral hemispheres inhibit each other through the corpus callosum fibers, maintaining a dynamic balance. After a unilateral stroke occurs, this balance is disrupted, with the UH becoming over- excited and inhibiting the AH (Bertolucci et al., 2018). The subsequent recovery process is closely related to the connections of the brain networks between the bilateral cerebral hemispheres (Swayne et al., 2008). Therefore, restoring the balance between the cerebral hemispheres is the key to functional recovery (Tang et al., 2015). MS-NIBS has significant advantages in achieving this balance. Five out of the six included studies effectively corrected the inter-hemispheric imbalance in inter-hemispheric competition by exciting the AH and inhibiting the UH. Only one study (Luo et al., 2022) used rTMS and tDCS to simultaneously stimulate the left DLPFC region. This might be because the benefits of rTMS in cognition mainly rely on DLPFC stimulation (Alcalá-Lozano et al., 2018). In addition, a study has found (Lee et al., 2016), that MS-NIBS can improve cognitive function in AD patients, especially at the mild/early stage of the disease, although no time × group interaction was observed. Another study showed (Nguyen et al., 2017) that long-term treatment (lasting more than 6 months) could clearly reduce the progression slope of cognitive decline in these patients. However, despite the fact that current studies have provided some insights into the benefits of MS-NIBS for patients with cognitive impairment, there are still significant knowledge gaps. Further observational studies incorporating multimodal imaging and neurophysiological techniques (Sale et al., 2015; Bergmann et al., 2016) are needed to: (1) validate whether MS-rTMS demonstrates superior long-term efficacy compared to conventional rTMS in targeted regions, and (2) elucidate the underlying neural mechanisms of the effects of MS-rTMS.

Despite the achievements of this study, there are also certain limitations: (1) The RCTs involve a series of subjective scales without objective indicators, which increases the heterogeneity of the indicators; (2) The number of reported studies is limited, and they are only from the recent 3 years, probably because this treatment strategy has been propos; (3) There are few foreign language literatures (only 2 out of 6 papers in this meta-analysis are from abroad), which may be related to the fact that MS-NIBS is not included in the latest guidelines for PSCI. Therefore, in the future, high-quality RCTs with multi-center, large-sample, and different combinations of stimulation parameters should be carried out to deeply explore the optimal application schemes of MS-NIBS at different durations after stroke. Meanwhile, research on the mechanism of action of MS-NIBS at different targets should be strengthened to promote the development of this field.

## 5 Conclusion

In terms of the overall cognitive function recovery, the treatment effect of the MS- NIBS group was significantly better than that of the SS-NIBS group. Moreover, MS- NIBS was superior to SS-NIBS in visuospatial ability and the trail making test. In addition, MS-TMS and the combined treatment of TMS and tDCS were more effective than single-site stimulation in the treatment of PSCI. This study has some limitations, and further exploration requires more objective metrics and optimal parameters to enhance its application.

## Data Availability

The original contributions presented in this study are included in this article/Supplementary material, further inquiries can be directed to the corresponding authors.

## References

[B1] Alcalá-Lozano R. Morelos-Santana E. Cortés-Sotres J. Garza-Villarreal E. Sosa-Ortiz A. González-Olvera J. (2018). Similar clinical improvement and maintenance after Rtms at 5 HZ using a simple vs. Complex protocol in Alzheimer’s disease. *Brain Stimul.* 11 625–627. 10.1016/j.brs.2017.12.011 29326021

[B2] Bai Y. (2024). *Observation on the rehabilitation efficacy of multi-site intermittent theta burst stimulation (iTBS) in the treatment of cognitive impairment after stroke.* Shijiazhuang: Hebei Medical University.

[B3] Bentwich J. Dobronevsky E. Aichenbaum S. Shorer R. Peretz R. Khaigrekht M. (2011). Beneficial effect of repetitive transcranial magnetic stimulation combined with cognitive training for the treatment of Alzheimer’s disease: A proof of concept study. *J. Neural Transm. (Vienna)* 118 463–471. 10.1007/s00702-010-0578-1 21246222

[B4] Bergmann T. Karabanov A. Hartwigsen G. Thielscher A. Siebner H. (2016). Combining non-invasive transcranial brain stimulation with neuroimaging and electrophysiology: Current approaches and future perspectives. *Neuroimage* 140 4–19. 10.1016/j.neuroimage.2016.02.012 26883069

[B5] Bertolucci F. Chisari C. Fregni F. (2018). The potential dual role of transcallosal inhibition in post-stroke motor recovery. *Restor. Neurol. Neurosci.* 36 83–97. 10.3233/rnn-170778 29439366

[B6] Borenstein M. Higgins J. Hedges L. Rothstein H. (2017). Basics of meta-analysis: I(2) is not an absolute measure of heterogeneity. *Res. Synth. Methods* 8 5–18. 10.1002/jrsm.1230 28058794

[B7] Cashin A. McAuley J. (2020). Clinimetrics: Physiotherapy evidence database (pedro) scale. *J. Physiother.* 66:59. 10.1016/j.jphys.2019.08.005 31521549

[B8] Chen S. Zhang X. Chen X. Zhou Z. Cong W. Chong K. (2023). The assessment of interhemispheric imbalance using functional near-infrared spectroscopic and transcranial magnetic stimulation for predicting motor outcome after stroke. *Front. Neurosci.* 17:1231693. 10.3389/fnins.2023.1231693 37655011 PMC10466792

[B9] Chen Y. Zhao Z. Huang J. Wang T. Qu Y. (2024). Computer-aided cognitive training combined with Tdcs can improve post-stroke cognitive impairment and cerebral vasomotor function: A randomized controlled trial. *BMC Neurol.* 24:132. 10.1186/s12883-024-03613-3 38641827 PMC11027365

[B10] Cho J. Lee A. Kim M. Park E. Chang W. Shin Y. (2017). Dual-mode noninvasive brain stimulation over the bilateral primary motor cortices in stroke patients. *Restor. Neurol. Neurosci.* 35 105–114. 10.3233/rnn-160669 28157112

[B11] Dacosta-Aguayo R. Graña M. Savio A. Fernández-Andújar M. Millán M. López-Cancio E. (2014). Prognostic value of changes in resting-state functional connectivity patterns in cognitive recovery after stroke: A 3t FMRI pilot study. *Hum. Brain Mapp.* 35 3819–3831. 10.1002/hbm.22439 24523262 PMC4282459

[B12] Di Pino G. Pellegrino G. Assenza G. Capone F. Ferreri F. Formica D. (2014). Modulation of brain plasticity in stroke: A novel model for neurorehabilitation. *Nat. Rev. Neurol.* 10 597–608. 10.1038/nrneurol.2014.162 25201238

[B13] Dowling N. Johnson S. Nadareishvili Z. (2024). Poststroke cognitive impairment and the risk of recurrent stroke and mortality: Systematic review and meta-analysis. *J. Am. Heart Assoc.* 13 e033807. 10.1161/jaha.123.033807 39239841 PMC11935622

[B14] Egger M. Davey Smith G. Schneider M. Minder C. (1997). Bias in meta-analysis detected by a simple, graphical test. *BMJ* 315 629–634. 10.1136/bmj.315.7109.629 9310563 PMC2127453

[B15] El Husseini N. Katzan I. Rost N. Blake M. Byun E. Pendlebury S. (2023). Cognitive impairment after ischemic and hemorrhagic stroke: A scientific statement from the American heart association/American stroke association. *Stroke* 54 e272–e291. 10.1161/str.0000000000000430 37125534 PMC12723706

[B16] Eriksson J. Nyberg L. Elgh E. Hu X. (2023). Improvement of cognition across a decade after stroke correlates with the integrity of functional brain networks. *Neuroimage Clin.* 37:103356. 10.1016/j.nicl.2023.103356 36842348 PMC9984887

[B17] Fang H. Gao F. Su B. Ren C. Jiang Z. (2022). Efficacy observation of bilateral transcranial direct current stimulation on visuospatial disorder in stroke patients. *Chin. J. Rehabil. Med.* 37 743–749. 10.3969/j.issn.1001-1242.2022.06.004

[B18] Feng Z. Deng X. Zhao N. Jing Y. Wang H. Zang Y. (2019). “Regulation of the function of attention-related brain regions by multi-target focused repetitive transcranial magnetic stimulation,” in *Proceedings of the 22nd national academic conference of psychology*, (Hangzhou).

[B19] Fischer D. Fried P. Ruffini G. Ripolles O. Salvador R. Banus J. (2017). Multifocal TDCS targeting the resting state motor network increases cortical excitability beyond traditional Tdcs targeting unilateral motor cortex. *Neuroimage* 157 34–44. 10.1016/j.neuroimage.2017.05.060 28572060 PMC12824506

[B20] Fitzgerald P. Benitez J. de Castella A. Daskalakis Z. Brown T. Kulkarni J. (2006). A randomized, controlled trial of sequential bilateral repetitive transcranial magnetic stimulation for treatment-resistant depression. *Am. J. Psychiatry* 163 88–94. 10.1176/appi.ajp.163.1.88 16390894

[B21] Fregni F. Boggio P. Valle A. Rocha R. Duarte J. Ferreira M. (2006). A Sham-controlled trial of a 5-day course of repetitive transcranial magnetic stimulation of the unaffected hemisphere in stroke patients. *Stroke* 37 2115–2122. 10.1161/01.STR.0000231390.58967.6b 16809569

[B22] Gao Y. Qiu Y. Yang Q. Tang S. Gong J. Fan H. (2023). Repetitive transcranial magnetic stimulation combined with cognitive training for cognitive function and activities of daily living in patients with post-stroke cognitive impairment: A systematic review and meta-analysis. *Ageing Res. Rev.* 87:101919. 10.1016/j.arr.2023.101919 37004840

[B23] Grimaldi G. Oulad Ben Taib N. Manto M. Bodranghien F. (2014). Marked reduction of cerebellar deficits in upper limbs following transcranial cerebello-cerebral Dc stimulation: Tremor reduction and re-programming of the timing of antagonist commands. *Front. Syst. Neurosci.* 8:9. 10.3389/fnsys.2014.00009 24523678 PMC3906576

[B24] Guo H. Z. Yu T. Wang L. Wu H. (2022). Research progress of transcranial magnetic stimulation based on different brain regions in the treatment of Alzheimer’s disease. *Pract. Geriatr.* 36 959–962.

[B25] Hara T. Shanmugalingam A. McIntyre A. Burhan A. (2021). The effect of non-invasive brain stimulation (Nibs) on attention and memory function in stroke rehabilitation patients: A systematic review and meta-analysis. *Diagnostics (Basel)* 11:227. 10.3390/diagnostics11020227 33546266 PMC7913379

[B26] Higgins J. Thompson S. (2002). Quantifying heterogeneity in a meta-analysis. *Stat. Med.* 21 1539–1558. 10.1002/sim.1186 12111919

[B27] Higgins J. Thompson S. Deeks J. Altman D. (2003). Measuring inconsistency in meta-analyses. *BMJ* 327 557–560. 10.1136/bmj.327.7414.557 12958120 PMC192859

[B28] Hu A. Huang C. He J. Wu L. (2023). Effect of repetitive transcranial magnetic stimulation combined with transcranial direct current stimulation on post-stroke dysmnesia: A preliminary study. *Clin. Neurol. Neurosurg.* 231:107797. 10.1016/j.clineuro.2023.107797 37263069

[B29] Huang Y. Chen S. Leng X. Kuo K. Wang Z. Cui M. (2022). Post-stroke cognitive impairment: Epidemiology, risk factors, and management. *J. Alzheimers Dis.* 86 983–999. 10.3233/jad-215644 35147548

[B30] Khedr E. Shawky O. Tohamy A. Darwish E. El Hamady D. (2012). Effect of anodal versus cathodal transcranial direct current stimulation on stroke recovery: A pilot randomized controlled trial. *Eur. J. Neurol.* 19:185. 10.1177/1545968313484808 23609526

[B31] Kim B. Kim D. Chun M. Yi J. Kwon J. (2010). Effect of repetitive transcranial magnetic stimulation on cognition and mood in stroke patients: A double-blind, sham-controlled trial. *Am. J. Phys. Med. Rehabil.* 89 362–368. 10.1097/PHM.0b013e3181d8a5b1 20407301

[B32] Klomjai W. Katz R. Lackmy-Vallée A. (2015). Basic principles of transcranial magnetic stimulation (TMS) and repetitive TMS (Rtms). *Ann. Phys. Rehabil. Med.* 58 208–213. 10.1016/j.rehab.2015.05.005 26319963

[B33] Lee J. Choi B. Oh E. Sohn E. Lee A. (2016). Treatment of Alzheimer’s disease with repetitive transcranial magnetic stimulation combined with cognitive training: A prospective, randomized, double-blind, placebo-controlled study. *J. Clin. Neurol.* 12 57–64. 10.3988/jcn.2016.12.1.57 26365021 PMC4712287

[B34] Lefaucheur J. Aleman A. Baeken C. Benninger D. Brunelin J. Di Lazzaro V. (2020). Evidence-based guidelines on the therapeutic use of repetitive transcranial magnetic stimulation (Rtms). *Clin. Neurophysiol.* 131 474–528. 10.1016/j.clinph.2019.11.002 31901449

[B35] Long H. Wang H. Zhao C. Duan Q. Feng F. Hui N. (2018). Effects of combining high- and low-frequency repetitive transcranial magnetic stimulation on upper limb hemiparesis in the early phase of stroke. *Restor. Neurol. Neurosci.* 36 21–30. 10.3233/rnn-170733 29439359

[B36] Luo Y. Chen J. Bai J. Chen S. Liu S. (2022). Efficacy of transcranial direct current stimulation combined with repetitive transcranial magnetic stimulation in the treatment of cognitive impairment after stroke. *Chin. J. Pract. Nerv. Dis.* 25 1203–1209. 10.12083/sysj.220974

[B37] Ma Q. Li R. Wang L. Yin P. Wang Y. Yan C. (2021). Temporal trend and attributable risk factors of stroke burden in China, 1990-2019: An analysis for the global burden of disease study 2019. *Lancet Public Health* 6 E897–E906. 10.1016/S2468-2667(21)00228-0 34838196 PMC9047702

[B38] Ma Y. Yang Y. Wang X. Huang Y. Nan J. Feng J. (2024). Prevalence and risk factors of poststroke cognitive impairment: A systematic review and meta-analysis. *Public Health Nurs.* 42 1047–1059. 10.1111/phn.13503 39702976

[B39] Maja S. (2013). *Cortical reorganisation of cerebral networks after childhood stroke: Impact on outcome.* Bern: Swiss National Science Foundation (SNF). 10.1186/s12883-015-0309-1 PMC4466862 26058895

[B40] Moher D. Liberati A. Tetzlaff J. Altman D. (2009). Referred reporting items for systematic reviews and meta-analyses: The prisma statement. *Ann. Intern. Med.* 151 264–9,w64. 10.7326/0003-4819-151-4-200908180-00135 19622511

[B41] Murad M. Wang Z. Chu H. Lin L. (2019). When continuous outcomes are measured using different scales: Guide for meta-analysis and interpretation. *BMJ* 364:k4817. 10.1136/bmj.k4817 30670455 PMC6890471

[B42] Nguyen J. Suarez A. Kemoun G. Meignier M. Le Saout E. Damier P. (2017). Repetitive transcranial magnetic stimulation combined with cognitive training for the treatment of Alzheimer’s disease. *Neurophysiol. Clin.* 47 47–53. 10.1016/j.neucli.2017.01.001 28161090

[B43] Park S. Koh E. Choi H. Ko M. H. (2013). A double-blind, sham-controlled, pilot study to assess the effects of the concomitant use of transcranial direct current stimulation with the computer assisted cognitive rehabilitation to the prefrontal cortex on cognitive functions in patients with stroke. *J. Korean Neurosurg. Soc.* 54 484–488. 10.3340/jkns.2013.54.6.484 24527190 PMC3921275

[B44] Polanía R. Nitsche M. Korman C. Batsikadze G. Paulus W. (2012). The importance of timing in segregated theta phase-coupling for cognitive performance. *Curr. Biol.* 22 1314–1318. 10.1016/j.cub.2012.05.021 22683259

[B45] Ren M. Xu J. Wang W. Shen L. Wang C. Liu H. (2024). Effect of dual-site non-invasive brain stimulation on upper-limb function after stroke: A systematic review and meta-analysis. *Brain Behav.* 14:e70145. 10.1002/brb3.70145 39508474 PMC11541860

[B46] Rizzo V. Siebner H. Morgante F. Mastroeni C. Girlanda P. Quartarone A. (2009). Paired associative stimulation of left and right human motor cortex shapes interhemispheric motor inhibition based on a Hebbian mechanism. *Cereb. Cortex* 19 907–915. 10.1093/cercor/bhn144 18791179

[B47] Sale M. Mattingley J. Zalesky A. Cocchi L. (2015). Imaging human brain networks to improve the clinical efficacy of non-invasive brain stimulation. *Neurosci. Biobehav. Rev.* 57 187–198. 10.1016/j.neubiorev.2015.09.010 26409343

[B48] Sasaki N. Kakuda W. Abo M. (2014). Bilateral high- and low-frequency Rtms in acute stroke patients with hemiparesis: A comparative study with unilateral high-frequency Rtms. *Brain Inj.* 28 1682–1686. 10.3109/02699052.2014.947626 25140931

[B49] Shepherd J. Huganir R. (2007). The cell biology of synaptic plasticity: AMPA receptor trafficking. *Annu. Rev. Cell. Dev. Biol.* 23 613–643. 10.1146/annurev.cellbio.23.090506.123516 17506699

[B50] Soleimani B. Dallasta I. Das P. Kulasingham J. Girgenti S. Simon J. (2023). Altered directional functional connectivity underlies post-stroke cognitive recovery. *Brain Commun.* 5:fcad149. 10.1093/braincomms/fcad149 37288315 PMC10243775

[B51] Sporns O. Chialvo D. Kaiser M. Hilgetag C. (2004). Organization, development and function of complex brain networks. *Trends Cogn. Sci.* 8 418–425. 10.1016/j.tics.2004.07.008 15350243

[B52] Swayne O. Rothwell J. Ward N. Greenwood R. (2008). Stages of motor output reorganization after hemispheric stroke suggested by longitudinal studies of cortical physiology. *Cereb. Cortex* 18 1909–1922. 10.1093/cercor/bhm218 18234688 PMC2474452

[B53] Tang Q. Li G. Liu T. Wang A. Feng S. Liao X. (2015). Modulation of interhemispheric activation balance in motor-related areas of stroke patients with motor recovery: Systematic review and meta-analysis of fMRI studies. *Neurosci. Biobehav. Rev.* 57 392–400. 10.1016/j.neubiorev.2015.09.003 26344667

[B54] Teasell R. W. Foley N. C. Bhogal S. K. Speechley M. R. (2003). An evidence-based review of stroke rehabilitation. *Top Stroke Rehabil* 10, 29–58. 10.1310/8yna-1yhk-ymhb-xte1 12970830

[B55] Tereshin A. Kiryanova V. Reshetnik D. Karyagina M. Konstantinov K. Lapin S. (2022). [The effect of non-invasive brain stimulation on neuroplasticity in the early recovery period after ischemic stroke]. *Vopr Kurortol Fizioter Lech Fiz Kult* 99 5–12. 10.17116/kurort2022990515 36279371

[B56] Valero-Cabré A. Amengual J. Stengel C. Pascual-Leone A. Coubard O. (2017). Transcranial magnetic stimulation in basic and clinical neuroscience: A comprehensive review of fundamental principles and novel insights. *Neurosci. Biobehav. Rev.* 83 381–404. 10.1016/j.neubiorev.2017.10.006 29032089

[B57] Valiengo L. Benseñor I. Goulart A. de Oliveira J. Zanao T. Boggio P. (2013). The sertraline versus electrical current therapy for treating depression clinical study (Select-Tdcs): Results of the crossover and follow-up phases. *Depress. Anxiety* 30 646–653. 10.1002/da.22079 23625554

[B58] Wang Y. Liu W. Chen J. Bai J. Yu H. Ma H. (2023). Comparative efficacy of different noninvasive brain stimulation therapies for recovery of global cognitive function, attention, memory, and executive function after stroke: A network meta-analysis of randomized controlled trials. *Ther. Adv. Chron. Dis.* 14:8754. 10.1177/20406223231168754 37332390 PMC10272674

[B59] Wang Y. Xu N. Wang R. Zai W. (2022). Systematic review and network meta-analysis of effects of noninvasive brain stimulation on post-stroke cognitive impairment. *Front. Neurosci.* 16:1082383. 10.3389/fnins.2022.1082383 36643019 PMC9832390

[B60] Woods A. Antal A. Bikson M. Boggio P. Brunoni A. Celnik P. (2016). A technical guide to TDCS, and related non-invasive brain stimulation tools. *Clin. Neurophysiol.* 127 1031–1048. 10.1016/j.clinph.2015.11.012 26652115 PMC4747791

[B61] Xu B. Gong Z. Wang X. Wang S. Wen W. Zhu H. (2022). Efficacy of alternating high and low frequency repetitive transcranial magnetic stimulation in the treatment of attention disorder after stroke. *J. Neurosci. Ment. Health* 22 275–280. 10.3969/j.issn.1009-6574.2022.04.009

[B62] Xu B. Lin C. Wang Y. Wang H. Liu Y. Wang X. (2024). Using dual-target Rtms, single-target Rtms, or sham Rtms on post-stroke cognitive impairment. *J. Integr. Neurosci.* 23:161. 10.31083/j.jin2308161 39207080

[B63] Yin M. Liu Y. Zhang L. Zheng H. Peng L. Ai Y. (2020). Effects of Rtms treatment on cognitive impairment and resting-state brain activity in stroke patients: A randomized clinical trial. *Front. Neural Circuits* 14:563777. 10.3389/fncir.2020.563777 33117131 PMC7561423

[B64] Zhang F. Qin Y. Xie L. Zheng C. Huang X. Zhang M. (2019). High-frequency repetitive transcranial magnetic stimulation combined with cognitive training improves cognitive function and cortical metabolic ratios in Alzheimer’s disease. *J. Neural Transm.* 126 1081–1094. 10.1007/s00702-019-02022-y 31292734

