# Supplementary material for: Effects of multi-site non-invasive brain stimulation on cognitive impairment after stroke: a systematic review and meta-analysis
Source: Front Hum Neurosci. 2025 Jun 30;19:1583566. doi: 10.3389/fnhum.2025.1583566 (PMC12256478; doi:10.3389/fnhum.2025.1583566)
Supplement: Supplementary file 1 [file Data_Sheet_1.doc]

**PUBMED**

(("Stroke"[Mesh]) OR (Strokes[Title/Abstract] OR Cerebrovascular Accident[Title/Abstract] OR Cerebrovascular Accidents[Title/Abstract] OR "CVA (Cerebrovascular Accident)"[Title/Abstract] OR "CVAs (Cerebrovascular Accident)"[Title/Abstract] OR Cerebrovascular Apoplexy[Title/Abstract] OR "Apoplexy, Cerebrovascular"[Title/Abstract] OR "Vascular Accident, Brain"[Title/Abstract] OR "Brain Vascular Accident"[Title/Abstract] OR "Brain Vascular Accidents"[Title/Abstract] OR "Vascular Accidents, Brain"[Title/Abstract] OR Cerebrovascular Stroke[Title/Abstract] OR Cerebrovascular Strokes[Title/Abstract] OR "Stroke, Cerebrovascular"[Title/Abstract] OR "Strokes, Cerebrovascular"[Title/Abstract] OR Apoplexy[Title/Abstract] OR Cerebral Stroke[Title/Abstract] OR Cerebral Strokes[Title/Abstract] OR "Stroke, Cerebral"[Title/Abstract] OR "Strokes, Cerebral"[Title/Abstract] OR "Stroke, Acute"[Title/Abstract] OR Acute Stroke[Title/Abstract] OR Acute Strokes[Title/Abstract] OR "Strokes, Acute"[Title/Abstract] OR "Cerebrovascular Accident, Acute"[Title/Abstract] OR "Acute Cerebrovascular Accident"[Title/Abstract] OR "Acute Cerebrovascular Accidents"[Title/Abstract] OR "Cerebrovascular Accidents, Acute"[Title/Abstract]))

AND ((("Cognition"[Mesh]) OR (Cognitions[Title/Abstract] OR Cognitive Function[Title/Abstract] OR Cognitive Functions[Title/Abstract] OR Cognitive Ability[Title/Abstract] OR Cognitive Abilities[Title/Abstract] OR Cognitive Performance[Title/Abstract] OR Cognitive Performances[Title/Abstract] OR Cognitive Functioning[Title/Abstract]) OR ("Attention"[Mesh] OR Attentions[Title/Abstract] OR Attentional Function[Title/Abstract] OR Attentional Functions[Title/Abstract] OR Attentional Ability[Title/Abstract] OR Attentional Abilities[Title/Abstract] OR Attentional Performance[Title/Abstract] OR Attentional Performances[Title/Abstract] OR Attentional Functioning[Title/Abstract])

OR ("Memory"[Mesh] OR Memories[Title/Abstract] OR Memory Function[Title/Abstract] OR Memory Functions[Title/Abstract] OR Memory Ability[Title/Abstract] OR Memory Abilities[Title/Abstract] OR Memory Performance[Title/Abstract] OR Memory Performances[Title/Abstract] OR Memory Functioning[Title/Abstract])

OR ("Executive Function"[Mesh] OR Executive Functions[Title/Abstract] OR Executive Functioning[Title/Abstract] OR Executive Ability[Title/Abstract] OR Executive Abilities[Title/Abstract] OR Executive Performance[Title/Abstract] OR Executive Performances[Title/Abstract]))

AND ((("Transcranial Direct Current Stimulation"[Mesh]) OR (tDCS[Title/Abstract] OR "Cathodal Stimulation Transcranial Direct Current Stimulation"[Title/Abstract] OR "Cathodal Stimulation tDCSs"[Title/Abstract] OR "Cathodal Stimulation tDCS"[Title/Abstract] OR "Stimulation tDCS, Cathodal"[Title/Abstract] OR "Stimulation tDCSs, Cathodal"[Title/Abstract] OR "tDCS, Cathodal Stimulation"[Title/Abstract] OR "tDCSs, Cathodal Stimulation"[Title/Abstract] OR Transcranial Random Noise Stimulation[Title/Abstract] OR Transcranial Alternating Current Stimulation[Title/Abstract] OR Transcranial Electrical Stimulation[Title/Abstract] OR "Electrical Stimulation, Transcranial"[Title/Abstract] OR "Electrical Stimulations, Transcranial"[Title/Abstract] OR "Stimulation, Transcranial Electrical"[Title/Abstract] OR "Stimulations, Transcranial Electrical"[Title/Abstract] OR Transcranial Electrical Stimulations[Title/Abstract] OR "Anodal Stimulation Transcranial Direct Current Stimulation"[Title/Abstract] OR "Anodal Stimulation tDCS"[Title/Abstract] OR "Anodal Stimulation tDCSs"[Title/Abstract] OR "Stimulation tDCS, Anodal"[Title/Abstract] OR "Stimulation tDCSs, Anodal"[Title/Abstract] OR "tDCS, Anodal Stimulation"[Title/Abstract] OR "tDCSs, Anodal Stimulation"[Title/Abstract] OR Repetitive Transcranial Electrical Stimulation[Title/Abstract]))

OR ("Transcranial Magnetic Stimulation"[Mesh] OR "Magnetic Stimulation, Transcranial"[Title/Abstract] OR "Magnetic Stimulations, Transcranial"[Title/Abstract] OR "Stimulation, Transcranial Magnetic"[Title/Abstract] OR "Stimulations, Transcranial Magnetic"[Title/Abstract] OR Transcranial Magnetic Stimulations[Title/Abstract] OR "Transcranial Magnetic Stimulation, Single Pulse"[Title/Abstract] OR "Transcranial Magnetic Stimulation, Paired Pulse"[Title/Abstract] OR "Transcranial Magnetic Stimulation, Repetitive"[Title/Abstract]))

EMBASE

| #1.((stroke:ab,ti OR strokes:ab,ti OR 'cerebrovascular accident':ab,ti OR 'cerebrovascular accidents':ab,ti OR cva:ab,ti) AND 'cerebrovascular accident':ab,ti OR cvas:ab,ti) AND 'cerebrovascular accident':ab,ti AND 'or cerebrovascular apoplexy':ab,ti OR 'apoplexy, cerebrovascular':ab,ti OR 'vascular accident, brain':ab,ti OR 'brain vascular accident':ab,ti OR 'brain vascular accidents':ab,ti OR 'vascular accidents, brain':ab,ti OR 'cerebrovascular stroke':ab,ti OR 'cerebrovascular strokes':ab,ti OR 'stroke, cerebrovascular':ab,ti OR 'strokes, cerebrovascular':ab,ti OR apoplexy:ab,ti OR 'cerebral stroke':ab,ti OR 'cerebral strokes':ab,ti OR 'stroke, cerebral':ab,ti OR 'strokes, cerebral':ab,ti OR 'stroke, acute':ab,ti OR 'acute stroke':ab,ti OR 'acute strokes':ab,ti OR 'strokes, acute':ab,ti OR 'cerebrovascular accident, acute':ab,ti OR 'acute cerebrovascular accident':ab,ti OR 'acute cerebrovascular accidents':ab,ti OR 'cerebrovascular accidents, acute':ab,ti |
| --- |
| #2.'transcranial direct current stimulation':ab,ti OR tdcs:ab,ti OR 'cathodal stimulation transcranial direct current stimulation':ab,ti OR 'cathodal stimulation tdcs':ab,ti OR 'cathodal stimulation tdcss':ab,ti OR 'stimulation tdcs, cathodal':ab,ti OR 'stimulation tdcss, cathodal':ab,ti OR 'tdcs, cathodal stimulation':ab,ti OR 'tdcss, cathodal stimulation':ab,ti OR 'transcranial random noise stimulation':ab,ti OR 'transcranial alternating current stimulation':ab,ti OR 'transcranial electrical stimulation':ab,ti OR 'electrical stimulation, transcranial':ab,ti OR 'electrical stimulations, transcranial':ab,ti OR 'stimulation, transcranial electrical':ab,ti OR 'stimulations, transcranial electrical':ab,ti OR 'transcranial electrical stimulations':ab,ti OR 'anodal stimulation transcranial direct current stimulation':ab,ti OR 'anodal stimulation tdcs':ab,ti OR 'anodal stimulation tdcss':ab,ti OR 'stimulation tdcs, anodal':ab,ti OR 'stimulation tdcss, anodal':ab,ti OR 'tdcs, anodal stimulation':ab,ti OR 'tdcss, anodal stimulation':ab,ti OR 'repetitive transcranial electrical stimulation':ab,ti OR 'transcranial magnetic stimulation':ab,ti OR 'magnetic stimulation, transcranial':ab,ti OR 'magnetic stimulations, transcranial':ab,ti OR 'stimulation, transcranial magnetic':ab,ti OR 'stimulations, transcranial magnetic':ab,ti OR 'transcranial magnetic stimulations':ab,ti OR 'transcranial magnetic stimulation, single pulse':ab,ti OR 'transcranial magnetic stimulation, paired pulse':ab,ti OR 'transcranial magnetic stimulation, repetitive':ab,ti  #3.'cognition':ab,ti OR 'cognitions':ab,ti OR 'cognitive function':ab,ti OR 'cognitive functions':ab,ti OR 'cognitive ability':ab,ti OR 'cognitive abilities':ab,ti OR 'cognitive performance':ab,ti OR 'cognitive performances':ab,ti OR 'cognitive functioning':ab,ti  #4.'attention':ab,ti OR 'attentions':ab,ti OR 'attentional function':ab,ti OR 'attentional functions':ab,ti OR 'attentional ability':ab,ti OR 'attentional abilities':ab,ti OR 'attentional performance':ab,ti OR 'attentional performances':ab,ti OR 'attentional functioning':ab,ti  #5.'executive function':ab,ti OR 'executive functions':ab,ti OR 'executive ability':ab,ti OR 'executive abilities':ab,ti OR 'executive performance':ab,ti OR 'executive performances':ab,ti OR 'executive functioning':ab,ti |

#6.'memory, working'/exp OR 'memory, working' OR 'working memory'/exp OR 'working memory' OR 'memory'/exp OR 'memory' OR 'long short term memory network'/exp OR 'long short term memory network' OR 'short term memory'/exp OR 'short term memory'

#7.#3 OR #4 OR #5 OR #6

#8.#1 AND#7

#9.#2 AND#8

**WEB OF SCIENCE**

TS=(Stroke OR Strokes OR Cerebrovascular Accident OR Cerebrovascular Accidents OR CVA (Cerebrovascular Accident) OR CVAs (Cerebrovascular Accident) OR Cerebrovascular Apoplexy OR Apoplexy, Cerebrovascular OR Vascular Accident, Brain OR Brain Vascular Accident OR Brain Vascular Accidents OR Vascular Accidents, Brain OR Cerebrovascular Stroke OR Cerebrovascular Strokes OR Stroke, Cerebrovascular OR Strokes, Cerebrovascular OR Apoplexy OR Cerebral Stroke OR Cerebral Strokes OR Stroke, Cerebral OR Strokes, Cerebral OR Stroke, Acute OR Acute Stroke OR Acute Strokes OR Strokes, Acute OR Cerebrovascular Accident, Acute OR Acute Cerebrovascular Accident OR Acute Cerebrovascular Accidents OR Cerebrovascular Accidents, Acute) AND TS=(Transcranial Direct Current Stimulation OR tDCS OR Cathodal Stimulation Transcranial Direct Current Stimulation OR Cathodal Stimulation tDCS OR Cathodal Stimulation tDCSs OR Stimulation tDCS, Cathodal OR Stimulation tDCSs, Cathodal OR tDCS, Cathodal Stimulation OR tDCSs, Cathodal Stimulation OR Transcranial Random Noise Stimulation OR Transcranial Alternating Current Stimulation OR Transcranial Electrical Stimulation OR Electrical Stimulation, Transcranial OR Electrical Stimulations, Transcranial OR Stimulation, Transcranial Electrical OR Stimulations, Transcranial Electrical OR Transcranial Electrical Stimulations OR Anodal Stimulation Transcranial Direct Current Stimulation OR Anodal Stimulation tDCS OR Anodal Stimulation tDCSs OR Stimulation tDCS, Anodal OR Stimulation tDCSs, Anodal OR tDCS, Anodal Stimulation OR tDCSs, Anodal Stimulation OR Repetitive Transcranial Electrical Stimulation OR Transcranial Magnetic Stimulation OR Magnetic Stimulation, Transcranial OR Magnetic Stimulations, Transcranial OR Stimulation, Transcranial Magnetic OR Stimulations, Transcranial Magnetic OR Transcranial Magnetic Stimulations OR Transcranial Magnetic Stimulation, Single Pulse OR Transcranial Magnetic Stimulation, Paired Pulse OR Transcranial Magnetic Stimulation, Repetitive) AND TS=(Cognition OR Cognitions OR Cognitive Function OR Cognitive Functions OR Cognitive Ability OR Cognitive Abilities OR Cognitive Performance OR Cognitive Performances OR Cognitive Functioning OR Attention OR Attentions OR Attentional Function OR Attentional Functions OR Attentional Ability OR Attentional Abilities OR Attentional Performance OR Attentional Performances OR Attentional Functioning OR Memory OR Memories OR Memory Function OR Memory Functions OR Memory Ability OR Memory Abilities OR Memory Performance OR Memory Performances OR Memory Functioning OR Executive Function OR Executive Functions OR Executive Ability OR Executive Abilities OR Executive Performance OR Executive Performances OR Executive Functioning) AND TS=(Randomized Controlled Trial OR controlled clinical trial OR random allocation OR double - blind OR single - blind OR Placebo OR Randomly OR randomized OR clinical trial* OR trial* OR RCT OR Random)

知网

(主题:卒中) OR (篇关摘:急性卒中+脑卒中+脑中风+脑血管意外+CVA+CVAs+脑血管中风+中风，急性+急性脑血管意外+急性脑卒中(精确)) AND (主题:认知) OR (篇关摘:认知+注意+记忆+执行(精确)) AND (主题:经颅直流电刺激) OR (篇关摘:经颅直流电刺激+经颅随机噪声刺激+经颅交流电刺激+经颅电刺激+阳极经颅直流电刺激+重复经颅电刺激+阴极经颅直流电刺激(精确)) OR (主题:经颅磁刺激) OR (篇关摘:经颅磁刺激+经颅磁刺激,单脉冲+经颅磁刺激,成对脉冲+经颅磁刺激,重复(精确))

万方

| (主题:(卒中) or 题名或关键词:(急性卒中 or 脑卒中 or 脑中风 or 脑血管意外 or CVA or CVAs or 脑血管中风 or 中风，急性 or 急性脑血管意外 or 急性脑卒中)) and (主题:(认知) or 题名或关键词:(注意 or 记忆 or 执行)) and (主题:(经颅直流电刺激 or 经颅磁刺激) or 题名或关键词:(经颅随机噪声刺激 or 经颅交流电刺激 or 经颅电刺激 or 阳极经颅直流电刺激 or 重复经颅电刺激 or 阴极经颅直流电刺激 or 经颅磁刺激, 单脉冲 or 经颅磁刺激, 成对脉冲 or 经颅磁刺激, 重复))  维普  题名或关键词=卒中 or 急性卒中 or 脑卒中 or 脑中风 or 脑血管意外 or CVA or CVAs or 脑血管中风 or 中风，急性 or 急性脑血管意外 or 急性脑卒中AND题名或关键词=认知 or 注意 or 记忆 or 执行AND题名或关键词=经颅直流电刺激 or 经颅随机噪声刺激 or 经颅交流电刺激 or 经颅电刺激 or 阳极经颅直流电刺激 or 重复经颅电刺激 or 阴极经颅直流电刺激 or 经颅磁刺激 or 经颅磁刺激,单脉冲 or 经颅磁刺激，成对脉冲 or 经颅磁刺激,重复  中国生物医学文献数据库  ("经颅直流电刺激"[常用字段:智能] OR "经颅随机噪声刺激"[常用字段:智能] OR "经颅交流电刺激"[常用字段:智能] AND "经颅电刺激"[常用字段:智能] OR "阳极经颅直流电刺激"[常用字段:智能] OR "重复经颅电刺激"[常用字段:智能] OR "阴极经颅直流电刺激"[常用字段:智能] AND "经颅磁刺激"[常用字段:智能]) AND ("认知"[常用字段:智能] OR "注意"[常用字段:智能] OR "记忆"[常用字段:智能] OR "执行"[常用字段:智能]) AND ("卒中"[常用字段:智能] OR "急性卒中"[常用字段:智能] OR "脑卒中"[常用字段:智能] OR "脑中风"[常用字段:智能] OR "脑血管意外"[常用字段:智能] OR "CVA"[常用字段:智能] OR "CVAs"[常用字段:智能] OR "脑血管中风"[常用字段:智能]) |
| --- |
